# Prevalence and antibiotic resistance pattern of urinary tract bacterial infections in Dessie area, North-East Ethiopia

**DOI:** 10.1186/1756-0500-7-687

**Published:** 2014-10-03

**Authors:** Asrat Agalu Abejew, Ayele A Denboba, Alemayehu Gashaw Mekonnen

**Affiliations:** Department of Pharmacy, College of Medicine and Health Sciences, P. O. Box: 1145, Dessie, Ethiopia; Department of Experimental Medicine and Surgery, Microbiology, Immunology and Infectious Disease program, Faculty of Medicine and Surgery, University of Rome Tor Vergata, Rome, Italy; Microbiology unit, Dessie Regional Health Research Laboratory, Dessie, Ethiopia

**Keywords:** UTI, Prevalence, Antibiotic, Susceptibility, Resistance

## Abstract

**Background:**

Different studies have indicated that urinary tract infections frequently occur in both community and hospital environments and are of the most common bacterial infections in humans. the outcomes of urinary tract infections are increased hospitalization, increased direct patient costs and mortality. In Dessie, the prevalence of the commmon pathogens and antibiotic susceptibility pattern is not well studied sofar. Thus, the aim of this study is to address these gaps in the study area.

**Methods:**

Retrospective study was conducted in Dessie regional health reseacrh laboratory from January 1-March 31, 2012. All culture and antibiotic susceptibility test results of patients’ diagnosed with UTI from September 2002 to September 2011 G.C were included in the study. Data were abstracted using structured questionnaires and finally, entered into SPSS Windows version 16.0, and descriptive statistics was generated to meet the study objective.

**Results:**

During the last ten years 680 (27.35%) bacteria were isolated in the regional laboratory. The most commonly isolated were *E. coli* 410 (60.29%), Pseudomonas species 59 (8.68%), Proteus species 53 (7.79%), S. *aurous* 50 (7.35%) and Klebsiella species 40 (5.88%). The *E.coli* were susceptible to Nitrofurantoin 43 (89.6%), furantoin 124 (87.3%), Nalidixic acid 91 (86.7%), kanamycin 116 (80%) & ciprofloxacin 66 (71.7%) but were almost resistant to Ampicillin, tetracycline, & trimethoprim-sulfamethoxazole. Similarly Pseudomonas and proteus species were resistant to almost all antibiotics except Gentamycin.

**Conclusion:**

The E.coli, pseudomonas and proteus species were the commonly isolated bacteria in the regional health research laboratory. A majority of isolated bacterial microbes were resistant to antibiotics commonly used in clinical practices and generally available in the local economy without prescription. Culture results are necessary before initiating antibiotics.

## Background

Urinary tract infections (UTI) are one of the most common bacterial infections in the human urinary system. Most of these infections involve the lower urinary tract, namely the bladder and the urethra. UTIs are much more common in elderly than younger individuals for a variety of reasons, and frequently occur both in community and hospital environments [1, 2]. Urethritis, cystitis, acute pylonephritis, prostatitis, and intra -renal and peri-nephric abscesses are the common types of UTIs [1–3]. The symptoms of UTI are non-specific and may lead to vague diagnosis [3, 4].

Women are at three times greater risk for UTI than men because of short, straight anatomy of the urethra, and termination of female urethra beneath the labia resulting in colonization by colonic gram-negative bacilli [1, 3, 5]. There are different pathogens that cause UTI [1, 3]. The *E.coli*, Klebsiella species, *P.aeruginosa*, and Enterococcus species were the most common bacterial pathogens isolated from the urinary tracts of infected patients [6–8]. UTIs are usually listed among common health care associated infections that occur mostly in health care facilities of low income countries due to cultural and environmental reasons [9–12].

The antibiotic sensitivity patterns of bacteria isolated from urinary tracts differ for different bacteria and antibiotics [6, 7, 13] with sensitivity to the Quinolones being usual while resistance is often seen to nitrofurantoin; Ampicillin and Cotrimoxazole. So far, *E.coli* has been reported as the most resistant microorganism almost for all antibiotics including Quinolones [6, 14] leading to recurrent infections [15, 16]. Irrational drug use such as long term use and low-dose antibiotic use due to lack of protocol for antibiotic use and empiric therapy due to lack of laboratory facility to determine sensitivity are the possible reasons for resistance and thus, recurrent infections and complicated UTIs [14–16].

Hence reporting common etiologic agents and respective antibiotic sensitivity is crucial for stakeholders to search for preventive and control measures against antibiotic resistance. There are few data regarding etiologic agents and respective antibiotic sensitivity in our country except limited studies on antimicrobial sensitivity. According to this study the overall prevalence of antibiotic resistance ranges from 65% to 72% [17, 18]. However, to our knowledge there was no published data on etiologic agents and antibiotic sensitivity in South Wollo. Thus, the aim of this study is to determine common etiologic agents and respective antibiotic sensitivity in an undeveloped country with some effective health care, as is common to many areas of the world.

## Methods

### Study area

This retrospective study was conducted in Dessie regional health research laboratory from January 1 to March 31, 2012 G.C. Dessie regional health research laboratory is located in Dessie town, Amhara region, North-East Ethiopia, 401 km from capital city, Addis Ababa. It is the only regional health research laboratory in North-East Amhara region serving laboratory requests from different areas of the region both governmental and non-governmental health organizations and from community too. According to Dessie town health administrative office, in the town there are 16 governmental health institutions (1 referral hospital, 1 primary hospital, 8 health centers and 6 health posts), and private health institutions (3 general hospitals, 6 higher clinics, 23 medium clinics, 15 pharmacies, and 24 drug stores).

### Study subjects

All patient data recorded on patient recording books from September 2002 to September 2011 G.C in the laboratory were included in the study. Similarly, the antibiotic sensitivity for the specific pathogen isolated was included in the study. Data were collected using a structured data collection format developed by the principal investigators. All the information including culture results and antibiotic sensitivity for isolated pathogens were collected.

### Laboratory diagnostic methods

The records data showed mid-stream (“clean catch”) urine specimen collected from each patient. Bacterial isolation and identification was performed according to standard operating procedures of Feingold and Martin [19]. Urine samples were inoculated on cystine lysine electrolyte deficient [CLED] agar and blood agar plates [Oxoid Basingstoke, UK] using standard wire loop [0.001 ml]. The inoculated agar plates were incubated at 37°C for 24 -48 hours. Then, the growths were inspected to identify the bacteria. Biochemical tests were performed on colonies from primary cultures for final identification of the isolates. In brief, Gram-negative bacteria were identified by performing a series of biochemical tests (Oxoid, LTD), namely triple sugar iron agar, indole, Simon’s citrate agar, lysine iron agar, urea, mannitol and motility. Gram-positive bacteria were identified based on their gram reaction, noviobiocin, catalase and coagulase test results.

The antimicrobial susceptibility tests of the isolates were performed according to the national committee for clinical laboratory standards (NCCLS) method using Kibry-Bauer disk diffusion test on Mueller-Hinton agar. Results were interpreted after measuring the zone of inhibition and comparing with the standards. *Eshercia coli* ATCC 25922 and S. aureus ATCC 25923 were employed as quality controls for the antimicrobial susceptibility test [20]. Finally data were edited, coded, and entered into SPSS Windows version 16.0, and descriptive statistics was computed to meet the stated objective.

### Ethical considerations

Ethical clearance was obtained from ethical review committee of Wollo University. The head of laboratory was requested for cooperation/permission by a formal letter from Wollo University. Patient privacy was protected by de-identification of records. Names of patients were replaced by initials. All data obtained in the course of the study were kept confidential, and used solely for the purpose of the study.

## Results

### Demographic characteristics of patients and tests performed

About 2486 culture results from urinary tract discharges and urine during the ten years period were included in the study. A majority of samples 1694 (68.1%) were from females, and 1373 (55.2%) were under age group of 16-35 years. Sex and age were unavailable for 34 (1.4%) and 218 (8.8%) of samples respectively (Table 1).

**Table 1:** **Sex distribution of patients in the Dessie regional health research laboratory, May, 2012**

S. No	Patient characteristics	Frequency (%)
	***Sex of patients***	
	Not mentioned	34 (1.4)
	Male	758 (30.5)
	Female	1694 (68.1)
	Total	**2486 (100)**
	***Age of patients***	
	Not mentioned	218 (8.8)
	<5 years	62 (2.5)
	5-15	151 (6.1)
	16-35	1373 (55.2)
	36-50	487 (19.6)
	> = 51	195 (7.8)
	**Total**	**2486 (100)**

Urine 2007 (80.7%) and vaginal discharge 395(15.9%) were the most commonly used samples for culture, while, urethral discharge being used in 84 (3.37%) cases.

**Figure 1:**
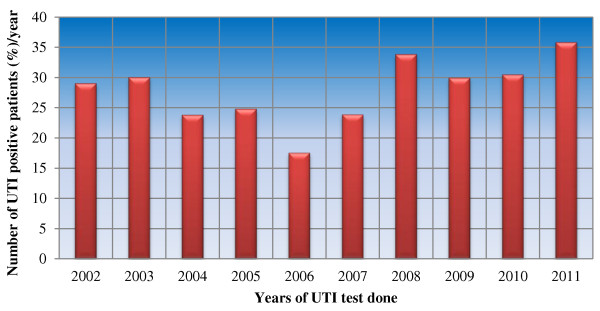
**Distribution of isolated bacteria per year in Dessie regional laboratory, May 2012.**

The number of samples cultured in each year tended to decrease over time. Thus, majority of samples were analyzed in between 2002-2004 G.C (12-14%), whereas, least number was during 2009-2011 G.C (5-8%). Majority of bacteria 71 (35.86%) were isolated in 2011 G.C but only 41 (17.52%) were isolated in 2006 G.C (Table 2, Figure 1).

**Table 2:** **Distribution of samples in years in Dessie regional health research laboratory, May, 2012**

S. No	Year	Samples (%)
1	2002	317 (12.8)
2	2003	323 (13.0)
3	2004	357 (14.4)
4	2005	266 (10.7)
5	2006	234 (9.4)
6	2007	285 (11.5)
7	2008	201 (8.1)
8	2009	177 (7.1)
9	2010	128 (5.1)
10	2011	198 (8.0)
**11**	**Total**	**2486 (100)**

### Prevalence and common types of bacterial uropathogens isolated

**Figure 2:**
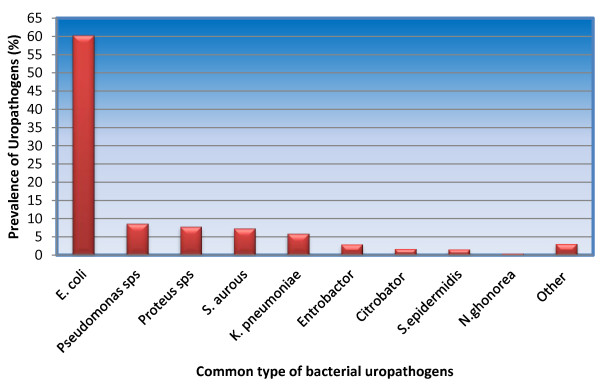
**Distribution of Isolated microorganisms in Dessie regional laboratory, May, 2012.**

About 680 (27.35%) bacteria were isolated from 2486 cultures. The most commonly isolated bacteria were *E. coli* 410 (60.29%) followed by Pseudomonas species 59 (8.68%), Proteus species 53 (7.79%), *S. aurous* 50 (7.35%) and *Klebsiella species* 40 (5.88%), respectively. Nevertheless, from the total positive samples of the patients about 21 (3.09%) of results were reported as ‘pathogenic microorganisms detected’, means the lab personnel were unable to identify the species of the microorganisms detected (Figure 2). About 528 (77.65%) and 372 (54.71%) of bacteria were isolated from females and in age group of 16-35 years respectively.

Approximately a quarter of the E.coli isolates were resistant to Amoxicillin, Ampicillin, tetracycline, and Trimetoprim-sulphamethoxazole (TMP-SMX) but were susceptible to Chloramphenicol, Ceftriaxone, Ciprofloxacin, Gentamicin, Kanamycin and Nalidixic acid. Antimicrobial susceptibility results are summarized in (Table 3).

**Table 3:** **Antibiotic Susceptibility pattern of isolated microorganisms in Dessie regional health research laboratory, May, 2012**

Antibiotic	***E. coli***		***Pseudomonas spps***		***Proteus spps***		***S. Aurous***		***Klebsiela spps***		***Entrobactor***		***S. epidermidis***		***N. gonorrhea***		***Citrobactor***	
	T _t_	%S	T _t_	%S	T _t_	%S	T _t_	%S	T _t_	%S	T _t_	%S	T _t_	%S	T _t_	%S	T _t_	%S
Amoxicillin	182	28(15.4)	8	1/8	14	0/14	3	1/3	20	6(30.0)	5	0/5	5	1/5	1	1/1	1	0/1
Tetracycline	314	56(17.8)	16	6(37.5)	23	6(26.1)	10	3/10	26	9(34.6)	19	8(42.1)	4	0/4	2	1/2	7	0/7
TMP-SMX	231	56(24.2)	27	9(33.3)	17	6(35.3)	15	4/15	23	8(34.8)	15	12/15	8	2/8	1	0/1	4	¾
Cephalothin	71	41(57.7)	8	0/8	10	3/10	2	1/3	5	5/5	7	2/7	5	2/5	1	0/1	2	½
Ampicillin	45	9(20.0)	3	1/3	4	0/4	1	0/1	8	1/8	1	0/1	1	0/1	2	2/2	1	0/1
Chloramphenicol	201	104(51.0	15	12/15	16	5(31.3)	7	4/7	21	13(61.9	19	13(68.4)	4	2/4	2	2/2	5	3/5
Ceftriaxone	196	104(53.1	15	10/15	14	614	14	8/14	23	11(47.8	15	11/15	2	2/2	2	2/2	3	2/3
Ciprofloxacin	92	66(71.7)	43	41(95.3)	10	9/1	18	11(61.1)	10	6/4	17	15(88.2)	4	¾	3	1/3	5	4/5
Doxycyclin	62	22(35.5)	10	2/10	7	2/7	3	2/3	7	4/7	1	1/1	-	-	-	-	1	0/1
Erythromycin	125	7(5.6)	39	7(17.9)	17	4(23.5)	27	10(37.0)	11	1/11	14	3/14	4	2/4	2	2/2	6	0/6
Gentamicin	362	239(66.0)	18	13(72.2)	25	16(64.0)	7	4/7	35	20(57.1)	19	16(84.2)	5	4/5	3	2/3	6	0/6
Kanamycin	145	116(80.0)	-	-	8	5/8	1	0/1	4	2/4	1	0/1	-	-	-	-	2	0/2
Naldixic acid	105	91(86.7)	-	-	8	5/8	-	-	9	9/9								
Nitrofurantoin	48	43(89.6)	4	4/4	4	4/4	3	3/3	6	4/6	6	5/6	-	-	1	1/1	1	1/1
Penicillin G	2	½	-	-	-	-	-	-	-	-	-	-	-	-	-	-	-	-
Vancomycin	31	3(9.7)	9	1/9	3	0/3	3	0/3	1	0/1	-	-	-	-	-	-	1	0/1
Furantoin	142	124(87.3)	1	0/1	13	5/13	1	1/1	24	16(66.7)			1	1/1			4	4/4
Carbencillin	127	42(33.1)	5	0/5	14	5/14	2	0/2	3	0/3	2	2/2	3	1/3			2	0/2
Clindamycin	26	16(61.5)	5	1/5	3	1/3	--	-	1	0/1	3	2/3	-	-	-	-	2	½
Bacitracin	45	13(28.9)	-	-	7	2/7	-	-	4	¼	-	-	1	0/1		-	-	-
Streptomycin	10	1/10	-	-	-	-	-	-	5	2/5	-	-	-	-	-	-	-	-
Oxacyclin	12	12/12	3	2/3	1	0/1	1	0/1	-	-	4	4/4	1	0/1	-	-	1	0/1

*Cut-off levels to establish sensitivity was done based on Oxoid the FDA susceptivity test interprative criteria available at: http://www.oxoid.com.

**Abbreviations *:* *T*_*t*_ Total tests, %*S* percent of sensitive results; for those samples ≤15 total sensitive/total tests (*T* ^***s***^ */T*_***t***_ ***)*** was used, *TMP-SMX*- trimetoprim-sulphamethoxazole, *spps* species.

## Discussion

According to this study out-off 680 [27.35%] cultures at least one bacterial species was isolated. The majority 528 (77.65%) were from females, and 372 (54.71%) from patients of 16-35 years old. This supports the idea women are more prone UTIs [1, 3, 5, 21]. Likewise, UTIs were more common among women of reproductive age groups (16-35 years) which agrees with earlier studies in this country and abroad in Nigeria, India and Kuwait [21–26] identified sexually active and/or probably pregnant females in this age group are at high risk for UTI.

The prevalence of isolated bacteria 680 [27.35%] were higher in this study compared with similar studies in the country [23 -25,36]. This might be due to data in this study were collected from culture results of patients referred for diagnostic purpose which will select for infected cases with gram-negatives 598 [87.94%] bacteria, which were most commonly isolated in this study. This prevalence differs with studies in other parts of the country [23, 24, 27] and other countries like Kuwait, Nigeria and Tanzania [6, 28, 29]. This might be due to geographical and/or methodological differences as well large site coverage, difference in source of data, laboratory verses hospitals, from other studies done in Ethiopia or other countries.

In this study like many other similar studies, *E. coli* remain the most prominent uropathogens which was isolated in 60.29% of the cultures. This is consistent with other studies in Ethiopia and abroad though the figure is slightly higher in this study [30–32]. Among the other frequently isolated bacterial uropathogens the most common were Pseudomonas species [8.68%], Proteus species [7.79%], *S.aurous* [7.35%] and *Klebsiella species* [5.88%]. This is similar when compared with other studies in Ethiopia and outside Ethiopia [23, 24, 27–29].

This study has showed that approximately a quarter of *E. coli* isolates were resistant to Amoxicillin, Ampicillin, Tetracycline, and Trimetoprim-sulphamethoxazole [TMP-SMX]. But were relatively sensitive to chloramphenicol, Ceftriaxone, Ciprofloxacin, Gentamycin, Kanamycin and Nalidixic Acid. This was partially in agreement with other studies in other parts of Ethiopia [23, 24, 27, 33] but was comparable with a report from Nigeria [6, 25]. The difference might be attributed to methodology, and pattern of management [34, 35]. In the study area there are many private pharmacies, hospitals and clinics which might have contributed to overuse of antibiotics and self-medication resulting in drug resistance as reported in earlier studies [14–16].

In comparison with the developed world the resistance rate of *E.coli* was elevated for all antibiotic treatments that have been used in the study area for the past ten years to treat UTIs [36–38]. For example, in Europe Amoxicillin resistance ranges 48% in Netherland to 60% in Belgium and from 21% to 36% for the TMP-SMX, whereas in our case it was 84.6% and 75.8%, respectively [39]. Hence, as it has been indicated in various researches the reason for difference might be factors related to poverty, although, difference in antibiotic use, patient population and prescribing rate can also play a role [36, 40–42].

In this study most isolates of Gram-negative bacteria were resistant to two or more drugs [multi-drug resistance] similarly a reported for studies in other parts of the country [24, 27]. This indicates that multi-drug resistance is increasingly becoming a major problem in the management of uropathogens in Ethiopia. This raises alarms to implement a nationwide antimicrobial surveillance and in-vitro susceptibility testing with strict adherence to antibiotic policy to inhibit the spread of drug resistant microbes in the country. Moreover, considering antibiotics with a resistance level of 10% or less are suitable for empiric therapy and the resistance level of antibiotics reported in this study were more than 10% for most common causative agents for UTI. Thus, these antibiotics are no longer appropriate for emparic management of UTIs. Hence, calls for nationwide study to know the exact level of antibiotics resistance among pathogenic bacteria is vital to make the right recommendation of alternative antibiotics and to support expert opinion [43].

As a limitation, this study may not represent general population that live in South Wollo area since only those who visited the health research laboratory either by referral or by patients own interest were included in the study. Only those antibiotics tested in the regional health research laboratory were included in the study. Thus, it may not include all antibiotics used in clinical practice. Being a retrospective study incomplete records and illegible hand writing eliminated some culture results and antibiotics from the study.

## Conclusion

In conclusion, the *E. coli*, pseudomonas and proteus species were the three commonly isolated microorganisms in the regional health research laboratory. Moreover, most isolated bacterial microbes were resistant to antibiotics used in clinical practices in the country. This calls for attention of health professionals and policy makers to consider the resistance pattern in their clinical practice, and policy making process respectively. Most importantly, these data may be used to control trends of antibiotic susceptibilities, to develop local antibiotic policies and to assist clinicians in the rational choice of antibiotic therapy and thus, to prevent indiscriminate use of antibiotics.

## References

[1] Dipiro JT, Talbert RL, Yee GC, Matzke GR, Wells BG, Posey LM (2011). Pharmacotherapy: A Pathphysiologic Approach.

[2] Bendall MJ (1984). A review of urinary tract infection in the elderly. J Antimicrob Chemother.

[3] Fauci AS, Brunwald E, Kasper DL, Longo DL, Hauser SL, Jameson JL, Loscallo J, Wiener C (2008). Disorder of the urinary and kidney tract. Harrison’s: Principles of Internal Medicine.

[4] Brien KO, Stanton N, Edwards A, Hood K, Butler CC (2011). Prevalence of Urinary Tract Infection (UTI) in sequential acutely unwell children presenting in primary care: exploratory study. Scand J Prim Health Care.

[5] Akinkugbe FM, Familusi FB, Akinkugbe O (1973). Urinary tract infection in infancy and early childhood. East Afr Med J.

[6] Kolawole AS, Kolawole OM, Kandaki-Olukemi YT, Babatunde SK, Durowade KA, Kolawole CF (2009). Prevalence of urinary tract infections (UTI) among patients attending Dalhatu Araf Specialist Hospital, Lafia, Nasarawa State, Nigeria. Int J Med Sci.

[7] Dias-Neto JA, Silva LDM, Martins ACP (2003). Prevalence and bacterial susceptibility of hospital acquired urinary tract infection. Acta Cir Bras.

[8] Khorvash F, Mostafavizadeh K, Mobasherizadeh S, Behjati M (2009). A Comparison of antibiotic susceptibility patterns of klebsiella associated urinary tract infection in spinal cord injured patients with nosocomial infection. Acta Med Iran.

[9] O’Neill E, Morris-Downes M, Rajan L, Fitzpatrick F, Humphreys H, Smyth E (2010). Combined audit of hospital antibiotic use and a prevalence survey of healthcare associated infection. Clin Microbiol Infect.

[10] Klevens RM, Edwards JR, Richards CLJ, Horan TC, Gaynes RP, Pollock DA, Cardo DM (2007). Estimating health care-associated infections and deaths in U.S. Hospitals, 2002. Public Health Rep.

[11] Memon BA (2007). Predominant and common cause of urinary tract infection(s) in Sukkur city. Rawal Med J.

[12] Warren DK, Zack JE, Mayfield JL, Chen A, Prentice D, Fraser VJ, Kollef MH (2004). The effect of an education program on the incidence of central venous catheter-associated bloodstream infection in a medical ICU. Chest J.

[13] Nwanze PI, Nwaru LM, Oranusi S, Dimkpa U, Okwu MU, Babatunde BB, Anake TA, Jatto W, Asagwara CE (2007). Urinary tract infection in Okada village: Prevalence and antimicrobial susceptibility pattern. Sci Res Essays.

[14] Aypak C, Altunsoy A, Düzgün N (2009). Empiric antibiotic therapy in acute uncomplicated urinary tract infections and fluoroquinolone resistance: a prospective observational study. Ann Clin Microbiol Antimicrob.

[15] Mahesh E, Ramesh D, Indumathi VA, Punith K, Kirthi R, Anupama HA (2010). Complicated urinary tract infection in a tertiary care center in South India. Al Ame en J Med Sci.

[16] Craig JC, Simpson JM, Williams GJ, Lowe A, Reynolds GJ, McTaggart SJ, Hodson EM, Carapetis JR, Cranswick NE, Smith G, Irwig LM, Caldwell PH, Hamilton S, Roy LP (2009). Antibiotic prophylaxis and recurrent urinary tract infection in children. N Engl J Med.

[17] Azene MK, Beyene BA (2011). Bacteriology and antibiogram of pathogens from wound infections at Dessie Laboratory, North East Ethopia [abstract]. Tanzania J Health Res.

[18] Abera B, Kibret M (2011). Bacteriology and antimicrobial susceptibility of otitis media at dessie regional health research laboratory, Ethiopia. Ethiop J Health Dev.

[19] Finegold SM, Martin WJ (1982). Diagnostic Microbiology.

[20] Bauer AW, Kirby WMM, Sherris JC, Turck M (1966). Antibiotic susceptibility testing by standard single disc method. Am J Clin Path.

[21] Griebling TL, Litwin MS, Saigal CS (2007). Urinary Tract Infection in Women. Urologic Disease in America.

[22] Jane-Francis TKA, Suylika Y, Njom HA, Esemu NS (2012). Etiologic profile and antimicrobial susceptibility of community-acquired urinary tract infection in two Cameroonian towns. BMC Res Notes.

[23] Beyene G, Tsegaye W (2011). Bacterial uropathogens in urinary tract infection and antibiotic susceptibility pattern in Jimma University Specialized Hospital, Southwest Ethiopia. Ethiop J Health Sci.

[24] Alemu A, Moges F, Shiferaw Y, Tafess K, Kassu A, Anagaw B, Agegn A (2012). Bacterial profile and drug susceptibility pattern of urinary tract infection in pregnant women at University of Gondar Teaching Hospital. Northwest Ethiopia. BMC Res Notes.

[25] Jombo GT, Emanghe UE, Amefule EN, Damen JG (2011). Urinary tract infections at a Nigerian university hospital: causes, patterns and antimicrobial susceptibility profile. J Microbiol Antimicrob.

[26] Mohammed A, Mohammed S, Asad UK (2007). Etiology and antibiotic resistance patterns of community-acquired urinary tract infections in J N M C Hospital Aligarh, India. Ann Clin Microbiol Antimicrob.

[27] Assefa A, Asrat D, Woldeamanuel Y, G/Hiwot Y, Abdella A, Melesse T (2008). Bacterial profile and drug susceptibility pattern of urinary tract infection in pregnant women at Tikur Anbessa Specialized Hospital Addis Ababa, Ethiopia. Ethiop Med J.

[28] Khalifa AB, Noura AS, Vincent OR (2010). Etiology and antibiotic susceptibility patterns of community and hospital-acquired urinary tract infections in a General Hospital in Kuwait. Med Princ Pract.

[29] Sabrina JM, Said A, Mabula K, Samuel YM (2010). Bacterial isolates and drug susceptibility patterns of urinary tract infection among pregnant women at Muhimbili National Hospital in Tanzania. Tanzania J Health Res.

[30] Fluit AC, Jones ME, Scharitz FJ, Acar J, Gupta R, Verhoef J (2000). Antimicrobial resistance among urinary tract infection (UTI) isolates in Europe: results from the SENTRY Antimicrobial Surveillance Program, 1997. Antonie von Leeuwenhock.

[31] El-Astal Z (2005). Bacterial pathogens and their antimicrobial susceptibility in Gaza Strip, Palestine. Pakistan J Med.

[32] Shaikh D, Ashfaq S, Shaikh K, Shaikh M, Naqvi BS, Mahmood ZA, Majid R (2005). Studies on resistance/sensitivity pattern of bacteria related with urinary tract infections. Med J Islamic Acad Sci.

[33] Biadglegne F, Abera B (2009). Antimicrobial resistance of bacterial isolates from urinary tract infections at Felge Hiwot Referral Hospital, Ethiopia. Ethiop J Health Dev.

[34] Pai V, Nair B (2012). Etiology and sensitivity of uropathogens in outpatients and inpatients with urinary tract infection: Implications on empiric therapy. Ann Trop Med Public Health.

[35] Khan AU, Zaman MS (2006). Multiple drug resistance patterns in urinary tract infection patients in Aligarh. Biomed Res.

[36] European Centre for Disease Prevention and Control (2010). Antimicrobial Resistance Surveillance in Europe 2009. Annual Report of the European Antimicrobial Resistance Surveillance Network (EARS-Net).

[37] Moffett SE, Frazee BW, Stein JC, Navab B, Maselli J, Takhar SS, Gonzales R (2012). Antimicrobial resistance in uncomplicated urinary tract infections in 3 California EDs. Am J Emerg Med.

[38] Khawcharoenporn T, Vasoo S, Ward E, Singh K (2012). High rates of Quinolones resistance among urinary tract infections in the ED. Am J Emerg Med.

[39] Van der Donk CF, Van de Bovenkamp JH, De Brauwer EI, De Mol P, Feldhoff KH, Kalka Moll WM, Nys S, Thoelen I, Trienekens TA, Stobberingh EE (2012). Antimicrobial resistance and spread of multi drug resistant Escherichia coli isolates collected from nine urology services in the Euregion Meuse-Rhine. PLoS One.

[40] Byarugaba DK (2004). Antimicrobial resistance in developing countries and responsible risk factors. Int J Antimicrob Agents.

[41] Nys S, Terporten PH, Hoogkamp-Korstanje JA, Stobberingh EE (2008). Trends in antimicrobial susceptibility of Escherichia co li isolates from urology services in The Netherlands (1998–2005). J Antimicrob Chemother.

[42] Rubin MA, Samore MH (2002). Antimicrobial use and resistance. Curr Infect Dis Rep.

[43] Gupta K, Hooton TM, Naber KG, Wullt B, Colgan R (2011). International clinical practice guidelines for the treatment of acute uncomplicated cystitis and pylonephritis in women: A 2010 update by the Infectious Diseases Society of America and the European Society for Microbiology and Infectious Diseases. Clin Infect Dis.

